# Is there an association between salivary immune and microbial profile with dental health in systematically healthy children?

**DOI:** 10.1007/s00784-024-05969-9

**Published:** 2024-10-03

**Authors:** Esti Davidovich, Hadar Sarne, Aviv Shmueli, David Polak

**Affiliations:** 1 Department of Pediatric Dentistry, Faculty of Dental Medicine, Hebrew University of Jerusalem - Hadassah Medical Center, Jerusalem, Israel; 2 Department of Periodontics, Faculty of Dental Medicine, Hebrew University of Jerusalem - Hadassah Medical Center, Jerusalem, Israel

**Keywords:** Salivary diagnostics, Cytokines, Saliva, Oral health, Caries, Gingivitis

## Abstract

**Objective:**

This study aimed to characterize the inflammatory profile of systemically healthy children’s saliva and its association with clinical diagnoses of caries and gingival inflammation.

**Materials and methods:**

Unstimulated saliva was collected from 100 children before clinical dental examinations. The saliva samples were analyzed for total protein and specific inflammatory cytokines (IL-10, IL-8, IL-6, and TNFα) with Bradford and ELISA assays, respectively. Salivary bacteria were quantified using a quantitative real-time polymerase chain assay. The salivary values were then correlated with age, DMFT index, plaque index (PI), and gingival index (GI).

**Results:**

The mean age of the cohort was 8.08 ± 0.23 years with 49% females, the mean DMF of the cohort was 2.64 ± 0.31, the mean GI was 0.51 ± 0.06, and the mean PI was 1.33 ± 0.07. Significant correlations were found between PI with DMFT and GI. Children with DMFT > 2 had significantly higher levels of IL-8 compared with children with DMFT ≤ 2. IL-6 and TNFα were significantly higher among children with PI > 1 than among children with PI ≤ 1.

**Conclusions:**

Salivary cytokine were found to be associate with clinical parameters as DMFT and PI, thus may be a potential tool that reflects dental health status.

**Clinical relevance:**

The presence of salivary cytokines in children may reflect evaluation of dental caries and oral inflammation.

**Supplementary Information:**

The online version contains supplementary material available at 10.1007/s00784-024-05969-9.

## Introduction

Saliva is a prodigious fluid that provides many, if not most, of the molecules found in the systemic circulation, rendering it a potentially valuable bio-fluid for diagnoses [1, 2]. Indeed, salivary diagnosis is a growing field, with identified markers associated with cancer, cardiovascular diseases, autoimmune diseases, viral and bacterial diseases, and human immunodeficiency [1]. One growing field in salivary diagnosis is its inflammatory profile as a potential marker for childhood illnesses, albeit with little attention to its profile in health and the impact of focal conditions, such as caries and gingivitis, on its composition [2–5].

Caries and gingivitis are common oral microbial conditions in systemically healthy children. Thus, the local oral environment in such conditions potentially affects focal inflammatory and microbial salivary cytokines. In such cases, salivary inflammatory and microbial profiles may alter and should be considered when using saliva to examine health and disease [2, 3].

Dental caries is associated with polymicrobial colonization on tooth surfaces. Both acidogenic and aciduric bacteria, such as the *mutans* group of Streptococci and Lactobacilli, are primary etiological agents of dental caries, and of those, *Lactobacilli* and *S. mutans* are mostly found together in saliva [6]. The diversity of microbes in saliva has been shown to increase the active status of caries [7]. Several types of inflammatory biomarkers are associated with oral diseases in saliva [3, 4]; Cytokines are among the most investigated biomarkers in this context [4] due to their involvement in inflammatory, infectious, and immunological diseases [4]. One salivary cytokine that has been studied in caries and gingivitis is interleukin-1 beta (IL-1β), indicating that it may be involved in the development of such conditions. Other studies have investigated the role of other salivary cytokines, such as tumor necrosis factor-alpha (TNFα) and interleukin-8 (IL-8), which are also involved in the immune response and contribute to the inflammatory process associated with caries and gingivitis. Nonetheless, the relationship between salivary cytokines and dental conditions in systemically healthy children has not been fully explored. Evidence shows that such diseases can be detected in adults through saliva biomarkers, such as interleukins IL-1β, IL-6, IL-8, and IL-10, TNFα, and matrix metalloproteinases (MMP)-8 and IL-9 [3, 4]. Susceptibility to dental caries will occur when there is an imbalance between oral microorganisms and the protective properties of saliva which, through cytokines, regulate the defense against cariogenic bacteria and modulate oral microbiota [8].

In gingivitis, bacteria such as *Streptococcus, Fusobacterium, Actinomyces, Veillonella, Treponema, Capnocytophaga,* and *Eikenella* [9] are involved. Taking this information into account, saliva may be used to measure bacterial load in such conditions.

In the literature there is no data connecting between salivary cytokines, bacteria caries and gingivitis in a systemically healthy children group at the same age.

The objective of this study was to characterize the inflammatory and microbiological profiles of the saliva of systemically healthy children and to associate those levels with a clinical diagnosis of caries and gingival disease. We hypothesize that dental conditions, such as caries and gingivitis, gravely affect salivary inflammatory and microbial profiles expressing in microbial counts and cytokines levels for each condition. Controlling for such confounders may be a mandatory step in the potential use of immunological salivary profiles as diagnostic tools.

## Materials and methods

### Study population

The study population comprised 100 children systemically healthy born during 2008–2016 (aged 4–12 years at the time of the study). Children with no previous relevant medical history and no medications on a regular basis. The participants were recruited between January 2020 and December 2022 from the Department of Pediatric Dentistry at the Hebrew University—Hadassah School of Dental Medicine. The inclusion criteria were systemically healthy children who did not take any medications and the children’s and their guardians’ agreement to participate in the research. The exclusion criteria, on the other hand, were children undergoing orthodontic treatment and children who did not fast before the saliva collection appointment.

The study was approved by the Hadassah Medical Center Helsinki Committee. (RMC-0204–14) All the participants’ parents signed informed consent forms.

### Clinical oral examination

Dental examinations were conducted by one dental student using a dental mirror, a dental explorer, and a dental probe. It included the following parameters:

- Oral hygiene – measured by the plaque index (PI) with a range of 0 (no plaque) to 3 (abundant plaque) and presented as an average for all sites, as described previously [10], only on buccal surfaces. (0- No plaque is in the area adjacent to the gingiva, 1-There is a plaque in the form of a thin film on the gingival margin, 2-There is a visible plaque in the gingival pocket and gingival margin, 3-There is a dense plaque in the gingival pocket and on the gingival margin).
- Periodontal status – measured by the gingival index (GI) using a periodontal probe and with a range of 0 (no bleeding) to 3 (spontaneous bleeding) and presented as an average for all sites, as described previously [11] (0-Healthy gums, 1-Mild discolouration and oedematous gingiva. No bleeding on probing, 2-Red, oedematous and shiny gingiva. There is bleeding on probing. 3- Red, oedematous and ulcerated gingiva. There is spontaneous bleeding).
- Caries status – measured by the DMFT/dmft index (D = decay, M = missing, F = filling; T per tooth) in permanent/ primary dentition [12, 13] and presented with a range of 0 (very low DMFT) to above 6.6 (very high DMFT) and presented as an average for all sites. All the values were collected and recorded in a data table.

### Saliva collection

Saliva was collected in a quiet room between 08:00 and 12:30 h. The children refrained from eating, drinking, brushing their teeth, or rinsing with mouthwash for at least 1 h before spitting. Immediately after clinical examination, the children were asked to collect saliva in their mouths and spit it into a sterile wide test tube for 3 min. The saliva was immediately stored at 4 °C without any additives and further kept at -80 °C until analysis. The data were collected on paper charts, which were transferred to a computer program (Excel, Microsoft, WA, USA).

### Bacteria testing

Bacterial DNA was extracted from saliva using a DNA extraction kit (Qiagen, Venlo, Netherlands). The DNA was then tested using specific primers for the total bacteria – *S. Mutans*, *Lactobacillus species,* and *Fusobacterium nucleatum* – using SYBR-Green-based quantitative real-time PCR (PCR Biosystems, London, UK) with the following protocol: a final volume of 20ul per group, all samples underwent the following thermal cycles – 95 °C for 2 min, 40 cycles of 95 °C for 10 s and 60 °C for 30 s, and a finish with 65 °C for 5 s [14–16]. The primer sets used in this study are shown in Supplementary Table 1. All reactions were carried out in duplicates, and all plates included a standard curve that was set in eight steps of tenfold dilutions of each bacteria that were used to standardize the results to absolute values.

### Salivary total protein quantification

The Bradford Coomassie Assay (BCA) was used to quantify the total salivary protein levels according to the manufacturer’s instructions (Pierce, Waltham, Massachusetts, United States). In brief, a standard curve was prepared using bovine serum albumin. All samples (standards and saliva samples) were then incubated with the BCA working reagent in a 96-well microplate at 37 °C for 30 min to allow color development that was measured at a 562 nm wavelength using a microplate reader.

### Salivary cytokine quantification

The salivary levels of human TNFα, IL-10, IL-8, and IL-6 were measured using ELISA kits according to the manufacturer’s instructions (R&D Systems, Minneapolis, MN, US). In brief, 96-well plates were coated with a carbonate-specific monoclonal antibody overnight at room temperature, blocked in 1% bovine serum albumin in phosphate-buffered saline (PBS), and tagged with a secondary detection antibody for 2 h at room temperature, followed by HRP conjugation and TMB as substrate. The reaction was then stopped with sulfuric acid and quantified at a 562 nm wavelength using a microplate reader.

### Statistical analysis

All experiments were performed in triplicates (for protein analysis) or duplicates (for PCR analysis). The data were stratified according to age, DMFT, PI, and GI. Three age groups were set according to dental age – primary dentition (ages 0–6 years), mixed dentition (ages 6–11 years), and permanent dentition (above age 11 years). DMFT was stratified by ≤ 2 and > 2. The PI was stratified by ≤ 1 and > 1, as a crud cutoff that allows clear differentiation when plaque is visible. GI was stratified by being equal to zero versus above zero, as such we have set the cutoff to 0.

The power calculation was set as the mean difference of salivary cytokine of 30 picograms between groups with a standard deviation of 20 picograms, alpha of 0.05, and 80% power, which resulted in groups of 16 subjects. Since the recruitment of children was done without knowing how many children would be in each stratification group (as stated above), the study recruitment limit was set at a total of 100 children.

The data were analyzed using a statistical software package (SigmaStat, Jandel Scientific, San Rafael, CA, USA). A one-way repeated measure analysis of variance (RM ANOVA) was applied to test the significance of the differences between the treated groups. When significant results were found, inter-group differences were tested for significance using Student’s t-test and the Bonferroni correction for multiple testing. The statistical significance was set at p < 0.05.

## Results

The study included 100 systemically healthy pediatric patients with a mean age of 8.08 ± 0.23, 49% of whom were female. 28 children were between 4–6 years old, 62 were between 6–11 and 10 were between 11–13 years old. There was no statistical difference between males and females. The mean DMFT for the cohort was 2.64 ± 0.31, the mean GI score was 0.51 ± 0.06, and the mean PI score was 1.33 ± 0.07.

### Stratification of clinical parameters according to age/PI/GI and DMFT

**Fig. 1:**
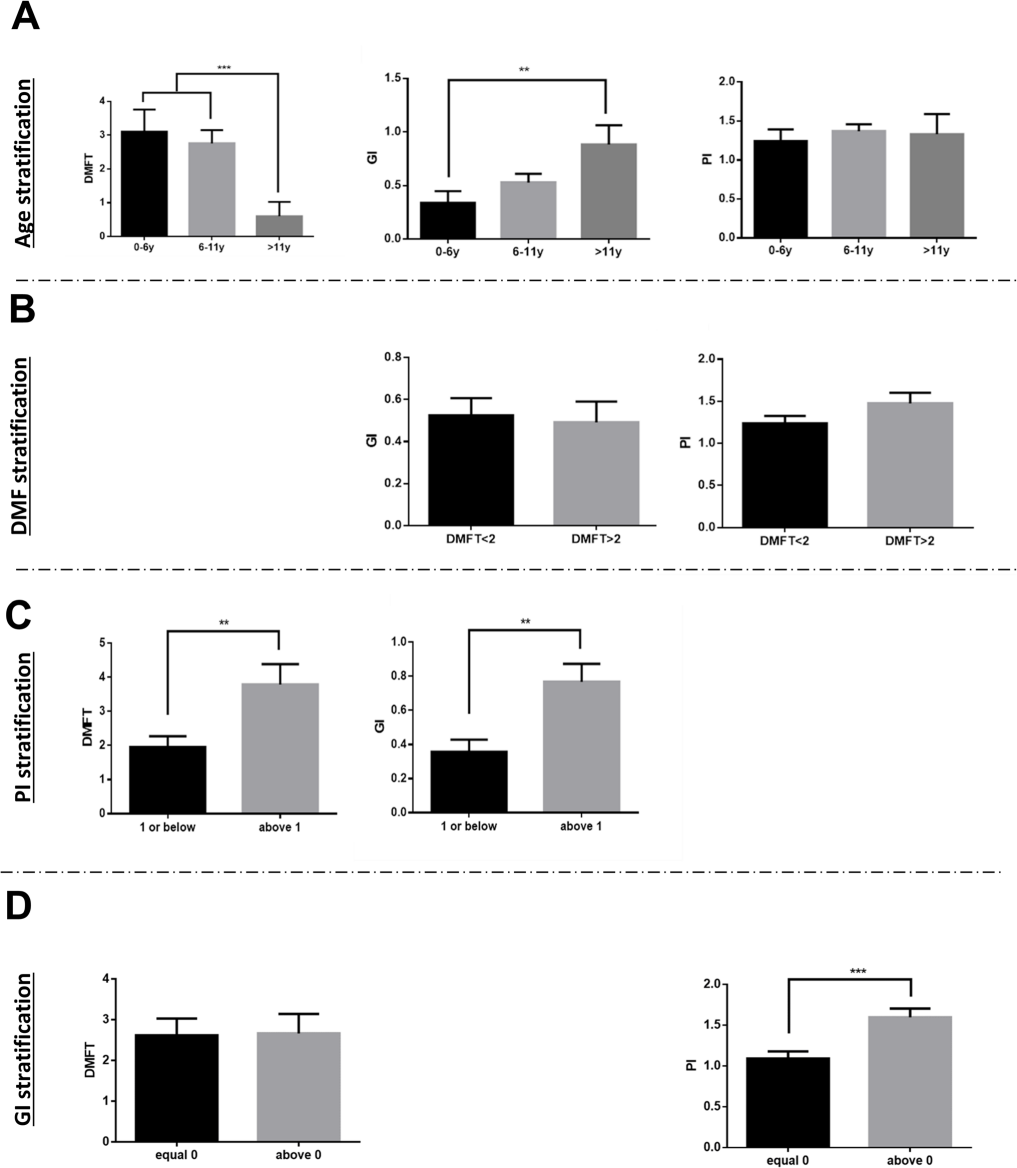
Stratification of clinical parameters according to age/PI/GI and DMF **A—**Mean and standard error of DMFT /PI/GI according to age group (0–6 years; 6–11 years and above 11 years). **B**—Mean and standard error of GI/PI according to DMFT groups (DMFT < 2 and DMFT > 2). **C**—Mean and standard error of DMFT /GI according to PI groups (1 or below and above 1). **D**—Mean and standard error of DMFT /PI according to GI groups (equal to 0 and above 0). **—*P* < 0.01; ***—*P* < 0.001

The mean DMFT level was significantly lower in the permanent dentition age group than in the other dental age groups (Fig. 1). The highest DMFT levels were observed for the primary dentition group (aged 6 years and younger), albeit without statistical difference compared with the mixed dentition group (Fig. 1). The mean GI level was statistically higher in the permanent dentition group than in the primary dentition group (Fig. 1). The mean PI did not differ substantially between the groups (Fig. 1, p = 0.14, r = 0.1).

GI and PI did not differ according to DMFT (Fig. 1). Significant associations were found of PI above 1 with DMFT and GI (Fig. 1). Significantly higher levels of PI were found among those with GI above 0 than among those with GI equal to zero (Fig. 1).

### Associations of caries and gingival inflammation with saliva proteins and salivary inflammatory cytokines

**Fig. 2:**
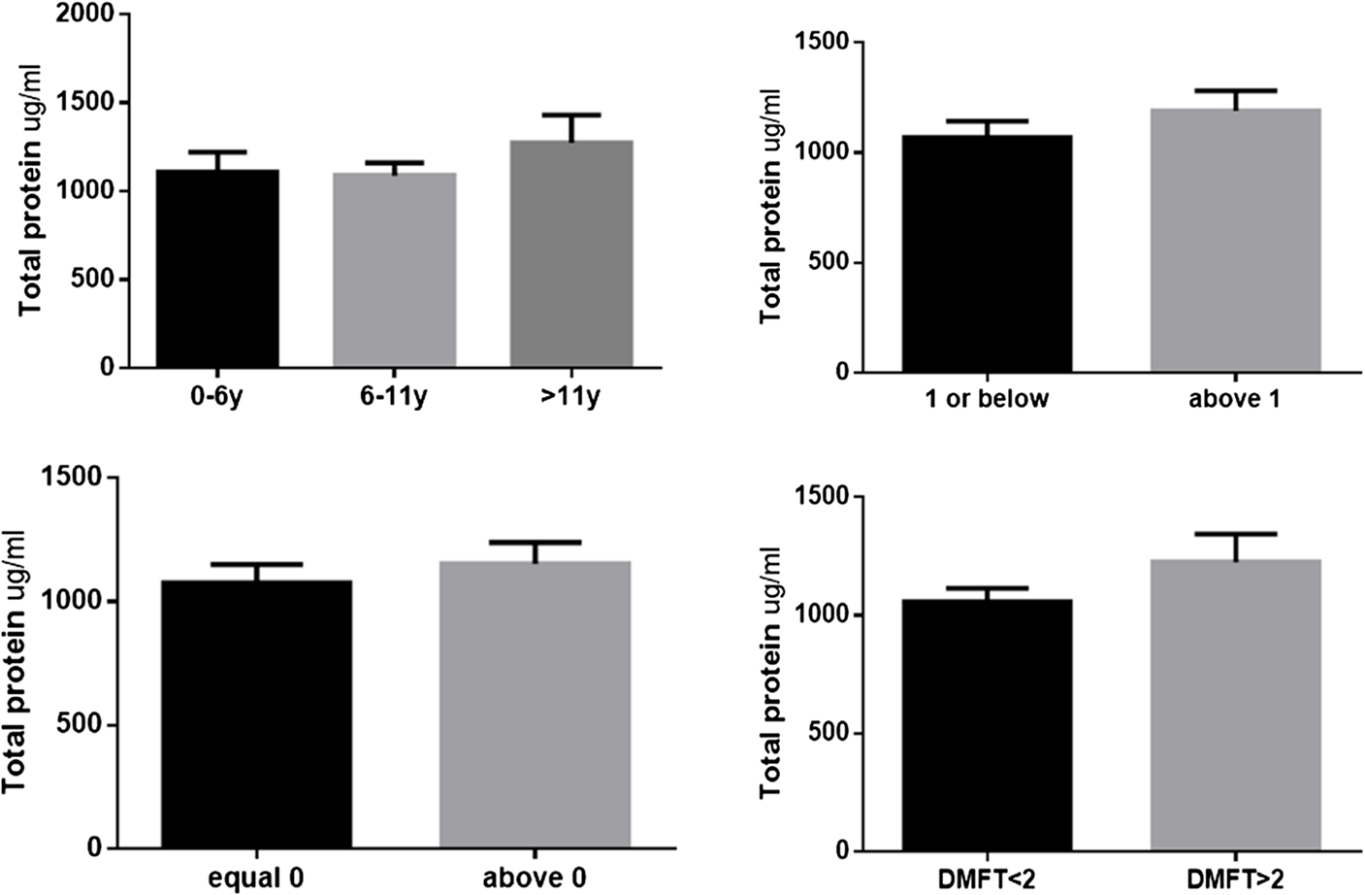
Associations of caries and gingival inflammation with saliva proteins. Mean and standard error of salivary protein levels (using BCA assay) according to age/ DMFT /PI and GI groups

The mean total protein level was higher, albeit without statistical significance, in the permanent dentition group than in the other dentition groups (Fig. 2). For those with DMFT > 2, GI above 0, and PI > 1 groups, protein levels were modestly higher than in the comparative groups, although these comparisons were without statistical significance (Fig. 2).

**Fig. 3:**
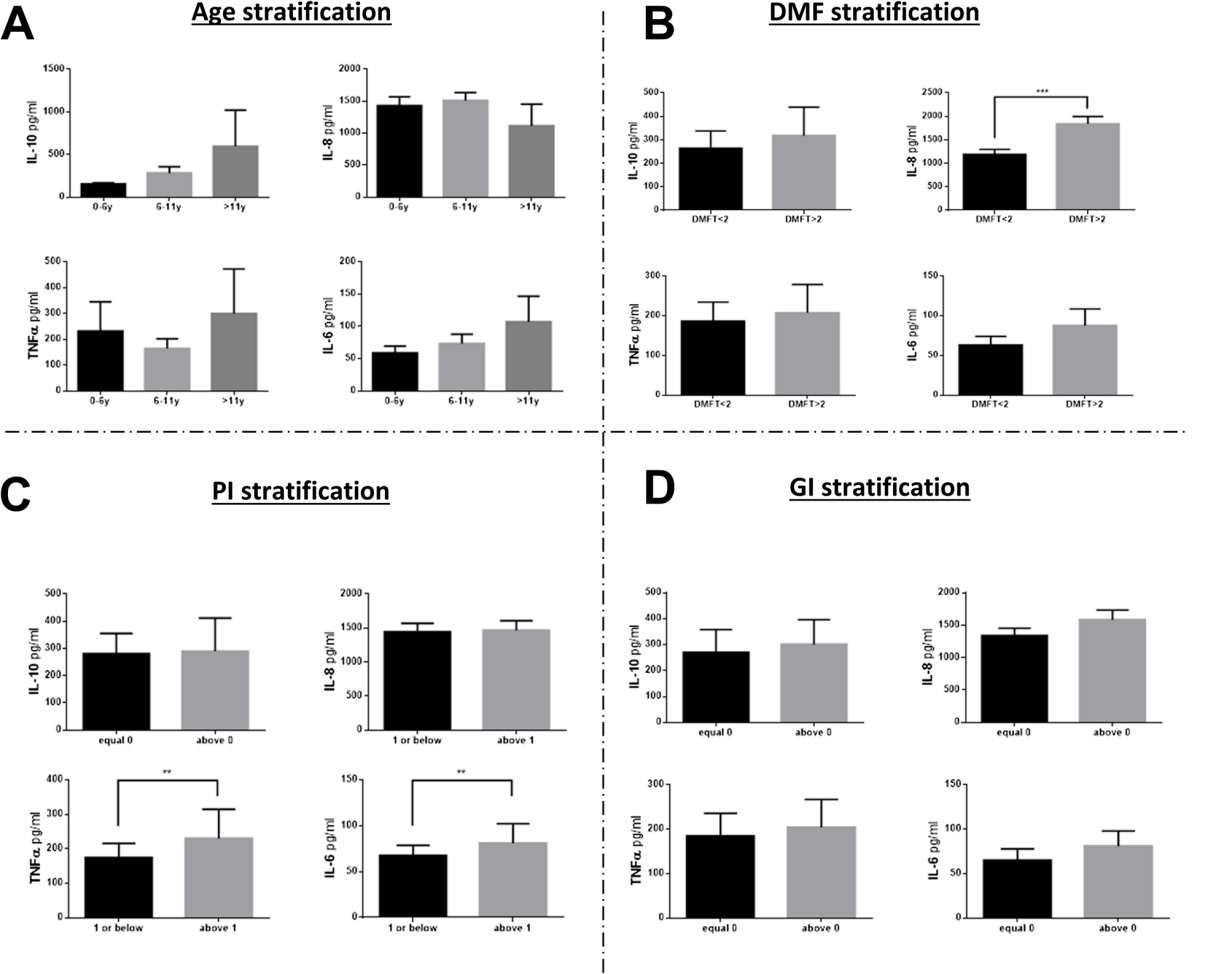
Associations of caries and gingival inflammation with saliva inflammatory cytokines. Mean and standard error of salivary cytokines (IL-10, TNFα, IL-8, and IL-6) levels (using ELISA) according to age (**A**), DMFT (**B**), PI (**C**), and GI (**D**) groups. **—*P* < 0.01; ***—*P* < 0.001

The levels of the inflammatory markers IL-10 and IL-6 showed a positive pattern according to age (Fig. 3) without any statistical differences. IL-8 and TNFα did not show age-dependent patterns (Fig. 3). The mean levels of the inflammatory markers examined were higher among those with DMFT > 2 than DMFT ≤ 2; the difference was statistically significant only for the IL-8 level (Fig. 3). IL-6 and TNFα were significantly higher among those with plaque > 1 than plaque ≤ 1 (Fig. 3). No statistically significant associations were observed between cytokine levels and GI (Fig. 3).

### Salivary bacterial load

**Fig. 4:**
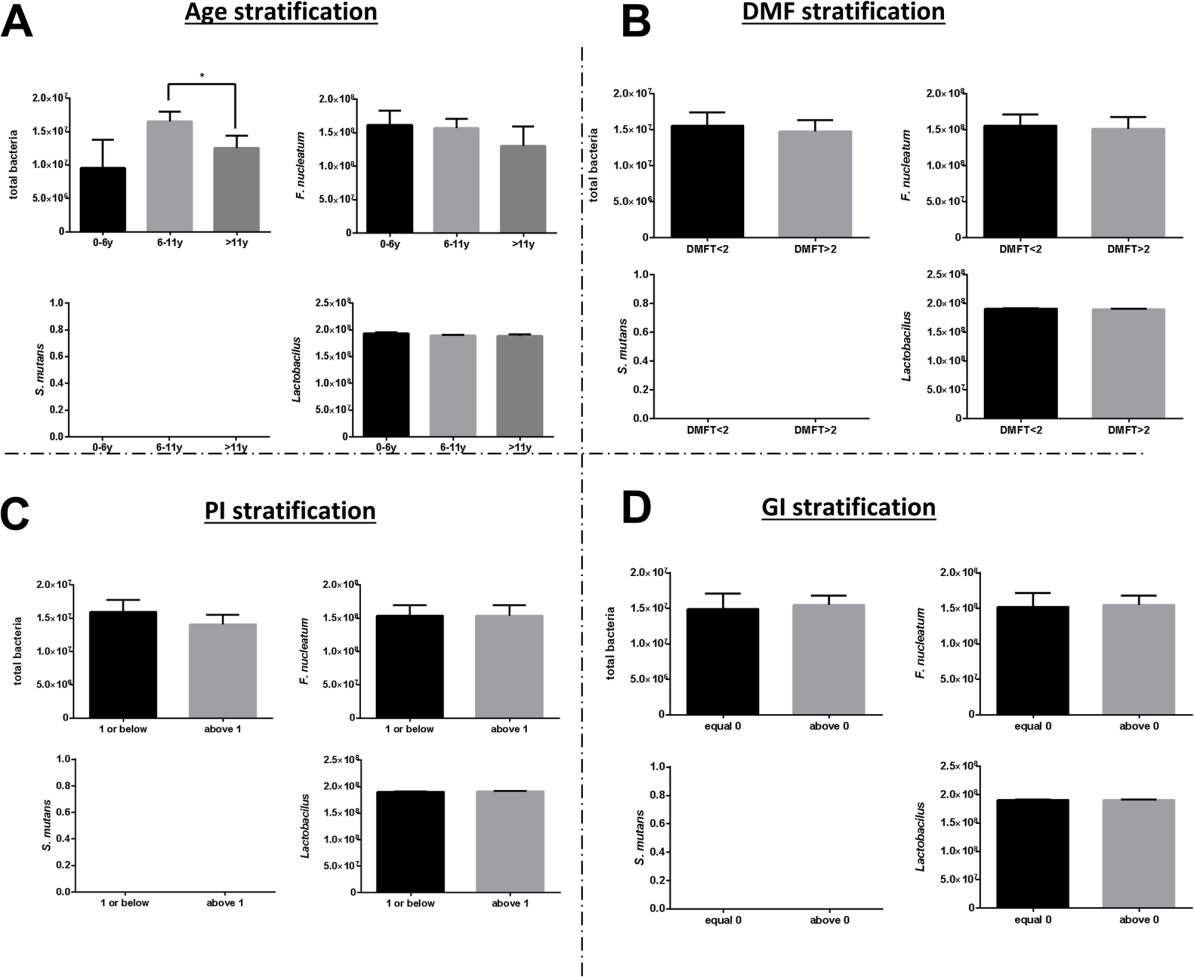
Associations of caries and gingival inflammation with salivary bacterial load. Mean and standard error of salivary bacteria (total bacteria, *F. nucleatum*, *S. mutans,* and *Lactobacillus*) levels (using RT-PCR) according to age (**A**), DMFT (**B**), PI (**C**), and GI (**D**) groups. *—*P* < 0.05

*S. Mutans* was undetectable in the saliva of all cases. The total bacterial load was significantly higher for the mixed dentition group than for the permanent dentition group (Fig. 4). The other stratifications did not show statistically significant differences in bacterial load (Fig. 4).

## Discussion

The main findings of the present study of healthy children are the significantly higher level of IL-8 among those with DMFT > 2 than DMFT ≤ 2, and the significantly higher mean IL-6 and TNFα levels among those with plaque > 1 than plaque ≤ 1. This highlights the impact of dental health status on saliva composition.

Several studies have reported associations between oral health status and various saliva properties in adults and children with systemic conditions [2, 5, 17–19]. In health saliva has numerous of functions: oral digestion, food swallowing and tasting, tissue protection and lubrication, maintenance of tooth integrity, and antibacterial and antiviral protection. Its inorganic components are accountable for osmotic balance, buffering capacity, and dental remineralization. Saliva’s main organic components are recognized as the first line of oral host defense. Saliva is considered a “mirror” of the body’s health or disease [20].

However, little is known about these properties in systemically healthy children. The findings of the current study may improve oral health diagnoses and may even improve the prevention and treatment of diagnosed conditions. To date, prospective longitudinal data on the prevalence of gingival inflammation in systemically healthy children’s oral cavities are scarce. In our study, children aged above 11 years (presumably with permanent dentition) showed a higher GI than younger children. This finding corroborates a study in which the presence of periodontal pathogens was common among children older than 6 years and peaked by 9–12 years [21].

While many studies have reported on the oral health status of preschool children [22, 23], only a few studies have targeted children with mixed dentition. The findings in this study may contribute to the development of oral health promotion strategies for children with mixed dentition. Our study also demonstrated that children with DMFT > 2 compared to those with DMFT ≤ 2 had significantly higher levels of IL-8. Previous studies have shown higher salivary levels of IL-8 among individuals with caries than without; however, some of those studies examined adults, while others examined DMFT values [24]. Moreover, in our study, the inflammatory markers, especially IL-6 and TNFα, were significantly higher among children with PI > 1 than with PI ≤ 1. Our results do not cohere with a study that did not find associations of IL-6 and TNFα with gingival clinical conditions in response to de novo plaque accumulation [19]. In a recent study, Yoshida et al., examined the association of cytokines with age, sex and dental state of children and found a positive association of IL-1β, IL-6, IL-8, and IL-10 with gingivitis [25], which cohere with the finding d of the current study (although the current data failed to show statistically significant differences). A similar pattern was also observed in cerebral palsy children [26]. Although there is a fair amount of data regarding salivary IL-1β, this cytokine was not measured in the current study due to the limited amount of saliva available for cytokine quantification. The fact that the PI was associated with salivary cytokines may stem from the fact that plaque levels are associated with gingivitis, and this leads to robust saliva inflammatory markers. The lack of correlation between GI and salivary inflammation may be due to low sensitivity of the GI versus PI or insufficient cases per group for a significance test.

Saliva contains many proteins that act directly or indirectly through various means on plaque and bacteria, modulating the tooth’s susceptibility to dental caries [27]. In our cohort, the total protein level did not differ significantly according to the DMFT values. The association between dental caries and total salivary protein has been examined in several studies, with contradictory findings. Some studies [28] found significantly higher protein levels in individuals with active caries than in caries-free controls, while other studies [22, 29] reported results similar to those of the current study. One study did not find a difference in total protein content according to DMF [27]. We report associations of a high PI score with GI and DMFT. Interestingly, a previous study demonstrated correlations between a high level of plaque and the levels of *S. Mutans* and *Lactobacillus* in children with severe early childhood caries compared with children without caries [19]. However, Inquimbert et al. interestingly found that bacteria known to be cariogenic did not present differences in abundance according to carious risk, they found that periodontal bacteria correlated with carious risk. They interestingly directed to estimation the risk of caries associated with bacterial factors in interdental sites of molars in adolescents as a better contributor to the definition of carious risk status [30].

Our study has several limitations. A larger cohort may have shown more prominent differences in cytokine levels; Dentition was considered by age group rather than by individual dental development; More cytokines should be examined; DMFT index does not measure active caries process but the history of disease; salivary flow rate was not examined.

In conclusion, we demonstrated associations of saliva composition with dental caries and gingival inflammation in systemically healthy children. The current study shows that saliva might be a potential tool for evaluating dental health status. This knowledge may promote earlier intervention and prevention among oral health professionals. Our results suggest that the presence of cytokines may be indicative and may serve as a tool for evaluating dental caries. To fully understand the ecological process in caries and gingival inflammation in children’s saliva, further molecular studies should involve not only cytokine identification and quantification but also metabolic and proteomic analysis. Furthermore, our approach and findings could be extended to studies that compare saliva results with other risk factors.

From a clinical point of view, one may argue that if salivary cytokines can be used as biomarkers for dental conditions, and thus can be used to establish a salivary test to detect early signs before their clinical manifestation; this is extremely important in prevention and treatment of systemically ill children. Moreover, such discoveries can be developed in the future as commercial kits for parents who wish to identify caries and gingivitis in their children.

## Bullet points

- Higher level of IL-8 among those with DMFT > 2 than DMFT ≤ 2.
- Higher levels of IL-6 and TNFα levels among those with plaque > 1 than plaque ≤ 1.
- The high presence of inflammatory cytokines may reflect dental caries status in children.
- These data provide a demonstration of the potential use of salivary cytokines in the oral cavity.

## Supplementary information

Below is the link to the electronic supplementary material.

Supplementary file1 (DOCX 31 KB)

Supplementary file2 (DOCX 13 KB)

## Data Availability

The data that support the findings of this study are not openly available due to reasons of sensitivity and are available from the corresponding author upon reasonable request. Data are located in controlled access data storage at department of pediatric dentistry Hadassah School of Dental Medicine.
