# Supplementary material for: Is there an association between salivary immune and microbial profile with dental health in systematically healthy children?
Source: Clin Oral Investig. 2024 Oct 3;28(10):564. doi: 10.1007/s00784-024-05969-9 (PMC11447006; doi:10.1007/s00784-024-05969-9)
Supplement: Supplementary file 2 — Supplementary file2 (DOCX 13 KB) [file 784_2024_5969_MOESM2_ESM.docx]

Supplementary table 1 - Primers design

| **Target gene** | **Forward** | **Reverse** | **Reference** |
| --- | --- | --- | --- |
| Universal | TCCTACGGGAGGCAGCAGT | GGACTACCAGGGTATCTAATCCTGTT | Dalwai et al., 2007 |
| *F. nucleatum* | AAGCGCGTCTAGGTGGTTATGT | TGTAGTTCCGCTTACCTCTCCAG | Dalwai et al., 2007 |
| *S. mutans* | AGCCATGCGCAATCAACAGGTT | CGCAACGCGAACATCTTGATCAG | Hata et al. 2006 |
| *Lactobacilus* | CTTGTACACACCGCCCGTCA | CTCAAAACTAAACAAAGTTTC | Shao et al., 2016 |
